# POU4F2/Brn-3b is Essential for Spermatogenesis and its Disruption is Linked to Male Infertility in Mice and Humans

**DOI:** 10.21203/rs.3.rs-7446280/v1

**Published:** 2025-10-31

**Authors:** Vishwanie Budhram-Mahadeo, Naomi Phillips, Anu Sironen, Mark Holt, Antoni Riera-Escamilla, Donald Conrad

**Affiliations:** University College London; University College London; University College London; King’s College London; Oregon Health & Science University, and Oregon National Primate Research Center; Oregon Health & Science University

**Keywords:** Male infertility, Spermatogenesis, Transcription factors, POU4F2/ Brn-3b, mutant mice

## Abstract

Male infertility is rising globally, yet its causes remain unclear. This study identifies the transcription factor Brn-3b (POU4F2) as essential for spermatogenesis and sperm function. Brn-3b is highly expressed in mature spermatids and infertility in constitutive male Brn-3b knockout (KO) mice is characterised by structural and functional testicular changes such as reduced sperm counts, impaired motility and ultrastructural defects including disrupted acrosomes and defects in the mitochondria and flagella. RNA-seq analyses reveal significant changes in Brn-3b-dependent regulation of genes essential for sperm development, mitochondrial function, and microtubule-based movement. This was confirmed using qRT-PCR with reduced expression of associated genes e.g. *Spata13, Dnah6*, Cox7a1 and upregulation of genes linked to inflammation and ECM remodelling (e.g., *Ptges, MMP2*). Human studies showing reduced Brn-3b in infertile men, e.g. with Klinefelter syndrome validated these findings. Exome sequencing identifying potentially deleterious variants in infertile men, suggest Brn-3b as a promising target for understanding and diagnosing male infertility.

## INTRODUCTION

The increasing prevalence of infertility, which now affects one in six couples globally, poses significant concerns for human populations(1, 2). Male factor infertility accounts for approximately 50–60% of cases, with impaired spermatogenesis and reduced sperm count being the most common contributing factors(3). A systematic review and meta-analysis have revealed a progressive and significant decline (> 50%) in sperm count among the general male population between 1973 and 2011(1). While lifestyle factors (e.g., obesity, smoking), environmental pollutants (e.g., plasticizers, bisphenol A [BPA]), and delayed parenthood may contribute to this trend, the precise molecular mechanisms remain unclear(4).

Throughout adulthood, male gametes are continuously produced in the seminiferous tubules from self-renewing primordial germ cells(5, 6). During spermatogenesis, diploid spermatogonia undergo a complex, highly regulated process involving meiotic division, differentiation, maturation, and remodelling, ultimately generating motile spermatozoa that are uniquely adapted to deliver genetic material to female gametes (oocytes) during fertilization(4–6). The specialized function of mature sperm is reflected in its complex and distinctive structure, with the sperm head containing the compact genetic material within the nucleus, covered by the cap-like acrosome that contains essential hydrolytic and proteolytic enzymes that are essential for oocyte fusion during fertilization(7, 8). Sperm motility relies on motor proteins within the dynamic tail flagellum that are fuelled by specialized mitochondria in the midpiece and energy derived from glycolysis in the fibrous sheath(4, 9).

These intricate processes are tightly regulated by changes in spatial and temporal gene expression in various cell populations including germ cells in the seminiferous tubules; Sertoli cells(6, 10), which support sperm production, and Leydig cells, which produce and secrete testosterone(6, 11). At a molecular level, transcriptional control of gene expression by tissue- and cell type specific DNA-binding transcription factors, is essential for mediating such diverse effects and dysregulation of such transcription factors is implicated in multiple human diseases (5, 12, 13). Understanding the regulatory factors that control spermatogenesis is crucial for pinpointing changes that affect sperm production or function and may contribute to infertility(3, 12).

Testes in fertile adult males continuously produce large volumes of highly specialised sperm cells throughout adult life and given the restricted size, each spermatozoon must express essential genes required for fertilization and early development(5, 13). Therefore, detection of POU4F2/Brn-3b (henceforth referred to as Brn-3b) in mature spermatids and sperm cells in mouse testes suggests previously unrecognized roles for this transcription factor in gene regulation and testicular function(14).

Brn-3b is a homeodomain transcription factor characterized by a highly conserved DNA-binding POU (Pit1; Oct1; Unc-86) domain, shared with other POU family members. Brn-3b proteins are highly conserved across species, exhibiting > 95% amino acid homology between human and mouse, highlighting important functions(15). Although initially isolated and cloned from neuronal cells, Brn-3b is widely expressed in diverse tissues, including reproductive tract tissue(14, 15). Brn-3b is a versatile transcription factor that regulate the transcription of diverse target genes by RNA polymerase II (RNAPII) either directly, by binding to unique DNA sequence sites (BRNF) in target gene promoters to activate or repress specific genes(15) or indirectly by binding to and modulating the transcriptional effects of other transcription factors such as the p53 tumour suppressor protein and the estrogen receptor (ER)(15–18). As such, can govern critical cellular processes such as proliferation, metabolism, differentiation, and apoptosis, depending on cell type and growth conditions(15, 17, 19, 20). For instance, Brn-3b enhances survival and differentiation in retinal ganglion cells (RGCs) by regulating key regulatory factors including *Sonic hedgehog (Shh)*, myostatin *(Gdf8)*, and *Pax4* and (21, 22). Yet, increased Brn-3b can drive proliferation in breast epithelial cells by activating cell cycle proteins, cyclin D1 and CDK4 while repressing the tumour suppressor gene, Brca1(23, 24). Brn-3b also controls cardiometabolic functions, by regulating distinct subsets of genes such as activating *Glut4* and repressing *Gsk3*β(20), which are key modulators of insulin sensitivity and glucose uptake in skeletal muscle, cardiac tissue, and adipose tissue. Brn-3b influences contractile function in vascular smooth muscle cells (VSMCs), by modulating calcium-dependent channels and pumps, including *Cacnd1d* Cacna1d (voltage-gated calcium channel), *Ryr2* (ryanodine receptor), and *Atp2a1/Serca1* (ATPase pump)(25). Consequently, loss of Brn-3b in leads to impaired glucose homeostasis consistent with type II diabetes (T2D), and also causes contractile dysfunction in the heart and vasculature in male mutant mice, due to dysregulated calcium signalling and sarco-endoplasmic reticulum stress(19, 25).

*Brn-3b* mRNA and protein expression in mouse testes, which was first documented in 2001, showed a significant and progressive increase in the maturing testis with highest levels in adult mouse testes, compared to the low levels detected in immature (juvenile) testes(14). Localization studies in sections of WT mouse testes showed Brn-3b expression primarily in mature spermatids at both the mRNA level (via in situ hybridization) and the protein level (via immunostaining). An important role for Brn-3b in the testis was also supported by studies showing that *Brn-3b* was undetectable in testes from infertile CREM knockout mice, which lack mature spermatids. Results from multiple human studies, including the Human Protein Atlas (HPA) project(26–28) have reported Brn-3b expression in the human testes and implicated key roles in male fertility(29–31). However, despite its unique expression in mature spermatids, the specific roles of this transcription factor during testes development or spermatogenesis are not fully explored. Importantly, homozygous male *Brn-3b* knockout (KO) mice are infertile, failing to produce any litters when mated with wild-type (WT), Brn-3b+/−, or Brn-3b−/− females. Therefore, the constitutive Brn-3b KO mutants were used to explore how *Brn-3b* loss affects testicular morphology and the mechanisms underlying infertility.

Herein, we show that constitutive loss of *Brn-3b* in mutant mice caused structural abnormalities in elongating spermatids linked to reduced sperm production and motility. Electron microscopy images identified clear ultrastructural defects in mutant spermatids with notable abnormalities in acrosomal structure, mitochondrial sheath and flagellum. Transcriptome analysis to compare testes gene expression from *Brn-3b* KO mutants and WT controls, revealed deregulation of genes associated with spermatogenesis, gamete generation, sperm motility, mitochondrial function and oxidative phosphorylation, as well as other signalling pathways. qRT-PCR validation studies also confirmed expression changes in selected genes required for spermatogenesis and sperm function including microtubule motor activity, electron transport chain and morphogenesis. Our results suggest novel and previously unknown functions for Brn-3b in fertile sperm production.

This was confirmed in human studies highlighting potential role(s) for *Brn-3b* in infertility in men. Single-cell RNA sequencing (scRNA-seq) revealed high *Brn-3b* expression in pachytene spermatocytes and elongating spermatids from fertile men, but significantly reduced expression in biopsies from infertile men. Additionally, whole exome sequencing (WES) also identified single-nucleotide variants (SNVs), including deleterious non-synonymous SNVs in infertile men with azoospermia and extreme oligozoospermia and one infertile man carrying two rare and predicted pathogenic variants in trans. Collectively, these results highlight the essential roles of *Brn-3b* in male fertility with potentially deleterious inactivation or loss of *Brn-3b* being associated with primary infertility.

## RESULTS

### Loss of *Brn-3b* affects seminiferous tubule diameter and spermatid differentiation:

Since *Brn-3b* expression was previously shown in mature spermatids of adult mouse testes^14^ and constitutive male *Brn-3b* mice are infertile, this model was used to investigate the impact of *Brn-3b* loss on testis morphology, spermatogenesis, and sperm structure.

**Figure 1:**
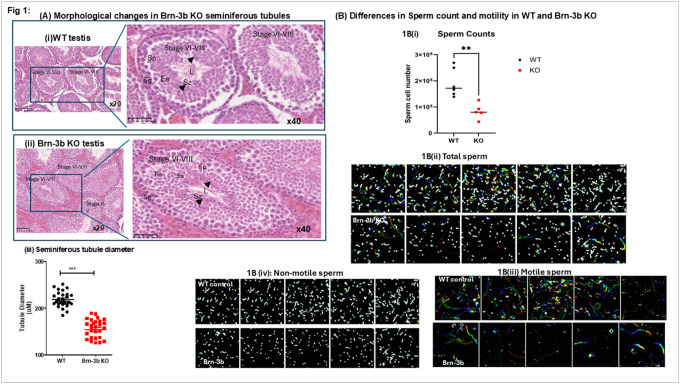
Comparison of morphology, sperm count and motility in Brn-3b KO mice compared with age-matched wild-type (WT) controls. (A) Representative images of cross sections of seminiferous tubules from (i) WT mice and (ii) Brn-3b KO mice, showing differences in morphological structure and cellular arrangement. Samples were stained with haematoxylin and eosin (H&E) and images were captured at x20 and x40 magnification, using Hamamatsu Nanozoomer imaging system. Arrowheads indicate spermatozoa (Sz) within the lumen (L). Sp - spermatocytes, Rs - round spermatids, Es- Early spermatids, and Sg indicates location of spermatogonia. (iii) Quantification of seminiferous tubule diameter was undertaken using measurements obtained in image J from H&E-stained sections. Data represents individual values of replicates as points, with mean shown as a line. Measurements taken from multiple tubules from at least 6 independent Brn-3b KO and WT testes. Statistical significance was determined using unpaired T-test. (B) (i) Infertility in male Brn-3b KO mice is associated in reduced sperm counts. Data represents total sperm counts per mouse as points, with mean shown as a line. WT n = 6; Brn-3b KO n = 5. Statistical significance was determined using an unpaired t-test. P = 0.003 presented as **. (ii-iv) Representative images showing relative motility of sperm taken from the epididymis of WT mice and Brn-3b KO mice at approximately 12 weeks of age. Image enhancement and segmentation were used to distinguish different populations of cells depending on positional changes, whereby non-motile cells were represented as white and highly motile cells appeared as a rainbow coloured ‘comet-tail’. (ii) Total cell population (iii) motile cells (iv) nonmotile cells.

To investigate whether loss of Brn-3b induces morphological or histological changes in the testes, we analysed multiple H&E-stained sections from independent wild-type (WT) and Brn-3b knockout (KO) mouse testes at varying magnifications. Figure 1A[i] presents representative cross-sectional images of stage VI–VIII seminiferous tubules from WT testis sections. At higher magnification, WT tubules exhibit a well-organized arrangement of germ cell populations with round, immature spermatogonia (Sg) located at the periphery of the tubules, with spermatocytes (Sc), round spermatids (Rs), elongating spermatids (Es) and mature spermatozoa (Sz) progressively positioned toward the lumen. Compact, elongated heads of spermatozoa are visible around the lumen (arrow), with long tails projecting into the lumen (Figure 1A[i], 40× magnification).

In contrast, Brn-3b KO testis sections show that despite a comparable distribution of immature spermatogonia and spermatocytes, there is a marked reduction in the number of elongated spermatids near the lumen (Figure 1A[ii], arrows) compared to WT controls.

We next analysed differences in seminiferous tubule diameter by measuring average tubule sizes across multiple sections from independent wild-type (WT) and *Brn-3b* knockout (KO) testes (see Methods). Figure 1A[iii] illustrates a significantly smaller tubule diameter in Brn-3b KO mutant testes compared to WT controls. This reduction may reflect a direct structural impact of loss of *Brn-3b* on seminiferous tubule structure, or alternatively, may represent a secondary change linked to diminished numbers of elongating spermatids within the tubules.

### Low sperm quality and motility in Brn-3b mutants:

We next tested if loss of Brn-3b affects sperm count and function by analysing samples collected from the dissected epididymides of age-matched wild-type (WT) and Brn-3b knockout (KO) mice. Sperm counts from independent WT (n=6) and *Brn-3b* KO (n=5) mice were plotted in a graph (Figure 1B[i]) or imaged and analysed for motility. Average sperm cell numbers, counted from independent WT and Brn-3b KO mice and shows that average sperm counts from Brn-3b KO mice were significantly lower than those from WT controls (unpaired t-test).

Time-lapse contrast-enhanced imaging of isolated live sperm (Fig. 1B[ii]) further confirmed the reduced sperm numbers in Brn-3b knockout (KO) mutants. Additional image processing was performed to distinguish motile from non-motile sperm cells, using colour enhancement to highlight positional changes in highly motile cells within each sample. Representative images in Figure 1B[iii], which highlight motile sperm cells, show a markedly higher number of motile cells in wild-type (WT) controls (top panel) compared to Brn-3b KO samples (lower panel). Non-motile cells, shown in Figure 1B[iv], also demonstrate reduced cell numbers in Brn-3b KO sperm samples, consistent with reduced sperm count.

These findings confirm that Brn-3b KO male mice produce fewer mature sperm cells in the epididymis, with a substantial reduction in motile sperm. This defect is highly likely to contribute to infertility observed in the mutant mice.

### Ultrastructural changes in elongated spermatids from *Brn-3b* KO mutants show disruption of sperm tail accessory structures and the acrosome:

**Figure 2:**
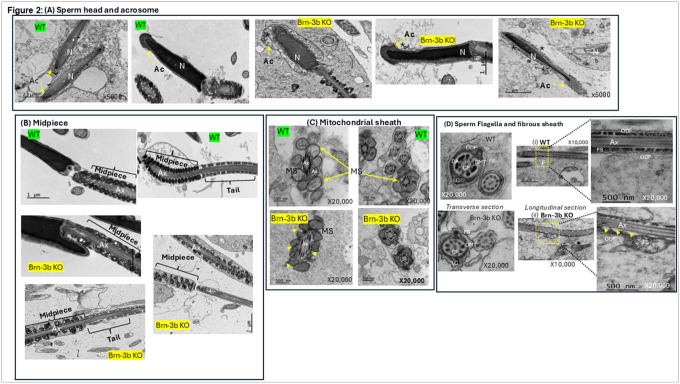
Transmission electron microscopy images showing key differences in Brn-3b/POU4F2 KO sperm samples compared with WT controls: Representative TEM images of sperm from wild-type or Brn-3b/POU4F2 KO mice showing changes in the (A) sperm head with asterix indicating potential damage to acrosome covering the sperm head in Brn-3b KO samples (B) mid-piece (C) mitochondrial sheath and (D) sperm flagella and the fibrous sheath. Magnification shown at x 5000-x 20,000. Ac = acrosome; Ax= axoneme; CP - central pair of singlet microtubules; DMT - microtubules doublets; FS- Fibrous sheath; LC- longitudinal columns; N = nucleus; MS = mitochondrial sheath; ODF – outer dense fibres.

TEM was used to analyse for ultrastructural changes in *Brn-3b* KO sperm, when compared with age-matched WT controls. Representative images in Fig 2, show that the most significant changes observed in *Brn-3b* KO spermatids were linked to the acrosome, midpiece and fibrous sheath. For instance, while WT spermatids displayed a compact head with condensed nucleus (N) surrounded by a well-defined acrosome (A) (Fig. 2A; top panels), comparable spermatids from *Brn-3b* KO displayed marked acrosomal changes, with membrane detachment and acrosomal fragmentation into multi-vesiculated and disrupted structures (Fig 2A; lower panels).

Significant structural changes are also observed in mid-piece (Fig 2B) with WT spermatozoa demonstrating the expected regular arrangement of compact mitochondria arranged within the well-defined mitochondrial sheath around the axoneme (Ax). In contrast, the mitochondrial sheath in the mid-piece of *Brn-3b* KO spermatids appeared less organised with smaller, less condensed mitochondria when compared to the mature WT mitochondria. Furthermore, with WT spermatids displaying a comprising intact, rounded, and well-organized mitochondria, which was contiguous with the well-organised principal piece of the flagellum.

Differences in mitochondrial organisation around the mid-piece are also highlighted in figure 2C where the mitochondrial content of the mid-piece in WT sperm displayed highly regular mitochondrial structures with defined membranes around the mitochondrial sheath while mid-piece from *Brn-3b* KO sperm, displayed smaller, more irregular mitochondrial structure with some evidence of fragmentation in mitochondrial content (arrowheads, lower panels).

Moreover, the adjoining fibrous sheath of the principal piece also appears disrupted. Furthermore, the principal piece of the flagella of sperm from *Brn-3b* KO mice also exhibited fibrous sheath (FS) dysplasia, with disruption and structural disorganization of the fibrous sheath and the outer dense fibres (ODF) when compared with the contiguous and intact fibrous sheaths and ODFs seen in control spermatids (Fig 2D).

These ultrastructural abnormalities in *Brn-3b* KO sperm, which include acrosomal defects, disorganization of the mitochondrial and fibrous sheath aligns with observation of infertility in *Brn-3b* KO male mice and may also explain our observation of reduced sperm count and poor motility in *Brn-3b* KO sperm.

### Deregulation of genes and pathways linked to male gamete generation, spermatogenesis, motility and bioenergetics in *Brn-3b* KO testes.

As a transcription factor, Brn-3b protein exerts diverse cellular effects by regulating the expression of specific target genes, in a highly tissue dependent manner. Therefore, infertility in male *Brn-3b* KO mice and the associated histological, functional and ultrastructural abnormalities in mutant sperm are likely to result from in changes in critical gene pathways that could provide insight into the molecular mechanisms for controlling spermatogenesis. As such, RNA sequencing analysis was next undertaken to identify genes that are differentially in testes from *Brn-3b* mutants when compared with WT controls.

**Figure 3:**
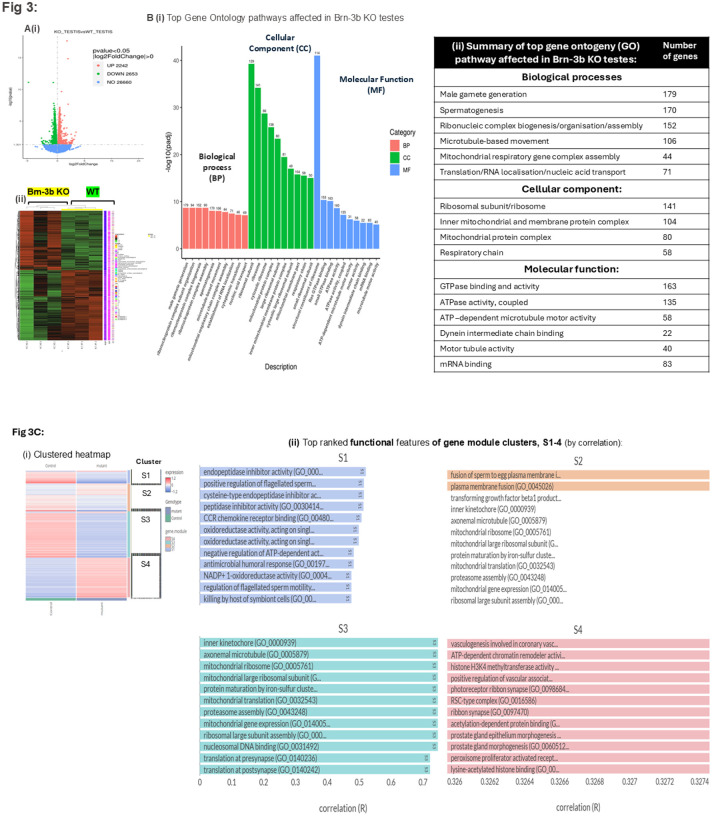
Summary of RNA sequencing data from Brn-3b KO testes compared with WT controls **A(i)**: Volcano plots showing the distribution of differentially expressed genes in testes form different Brn-3b KO vs WT control. From a total of >30,000 genes analysed 2242 genes were upregulated (red dots), 2653 genes down regulated and 26,660 unchanged genes. The x-axis shows the fold change in gene expression between different samples, and the y-axis shows the statistical significance of the differences. (ii) Heat Map showing hierarchical clustering of differentially expressed genes in the Brn-3b testis compared with WT controls. Red indicates genes with high expression levels, and green indicates genes with lower expression levels. The color ranging from red to blue indicates that log2 (FPKM+1). FPMK=Fragments Per Kilobase of transcript per Million. Upregulated genes are shown in red and down regulated genes in green. **(B)** (i) Top-ranked Top Gene Ontology pathways for Biological process (BP); Cellular Component (CC) and Molecular Function (MF), enriched for differentially regulated genes in Brn-3b KO testis, compared with age-matched WT controls. Table highlighting top Gene Ontology pathways affected in Brn-3b KO testes and classified into for Biological process (BP); Cellular Component (CC) and Molecular Function (MF) key biological processes. (ii) Summary of top Gene ontology pathways and number of genes affected in Brn-3b KO testes. **C (i)**: Data from BigOmics Analytics platform showing (i) clustered heat-map of S1-S4 of differentially regulated genes significantly altered in testes from Brn-3b KO mutants compared with WT controls. (ii) Functional annotation of top ranked features (by correlation) for gene module clusters, S1–4.

RNA extracted from independent testes of *Brn-3b* KO mice and WT controls was used for high throughput RNA sequencing and bioinformatics analyses. Figure 3A shows (i) the volcano plot and (ii) heat map of differentially expressed genes (p value <0.05), with 2242 upregulated genes and 2653 down regulated genes in KO testes when compared with WT controls. Gene Ontology (GO) analyses of differentially regulated genes highlight the top biological processes (BP), cellular components (CC) and molecular functions (MF) affected in *Brn-3b* KO testes when compared with WT controls (Fig 3B [i]). The number of genes associated with selected pathways are shown in Fig. 3B[ii]. The top biological processes affected by loss of Brn-3b in the testes were linked to male gamete generation (179 genes) and spermatogenesis (170 genes) highlighting key roles for Brn-3b in regulating such processes. The hierarchical network of enriched biological processes shown in the directed acyclic graph (DAG) (Supplementary Fig 1) shows that several nodes affected in mutant testes converge on biological processes associated with sexual reproduction, specifically male gamete generation (GO.004823276) and spermatogenesis (GO.0007283). The top genes associated with these pathways (Table 1) include families of proteins with known functions in spermatogenesis e.g. SPATA; DNAH and AKAP proteins.

Other biological processes affected in *Brn-3b* KO testes included ribonucleic complex biogenesis and assembly (152 genes), microtubule-based movement (106 genes) and mitochondrial respiratory chain complex assembly (44 genes). Analysis of the cellular components affected by loss of Brn-3b in testes, shows that in line with the biological processes, differentially regulated genes were associated with either the ribosomal complex or the mitochondrial complexes, including the respiratory chain suggesting key functions in protein translation and metabolic processes linked to oxidative phosphorylation and bioenergetics. These findings were also consistent with molecular functions, with differentially regulated genes involved in GTPase binding and activity and ATPase-coupled activities as well as cell motility (dynein chain binding and microtubule/motor tubule activity). This data suggests that loss of *Brn-3b* in the testes profoundly affects spermatogenesis and sperm function but also caused disruption in mitochondrial bioenergetics and motility related activities.

The effects of loss of *Brn-3b* on genes associated with specific gene ontology pathways was also confirmed by undertaking additional bioinformatics analyses using the BigOmics Analytics platforms^32^, which classifies differentially expressed genes into clusters based on top-ranked features (by correlation) and subsequent functional annotation of gene modules in each of the four clusters. The heat map of top-ranked differentially regulated genes, shown in figure 3C(i) highlights the overall changes in each cluster, with differentially expressed genes in clusters S1 and S3 being primarily down-regulated in *Brn-3b* KO testis while cluster S4 contained genes that were mainly upregulated in mutants when compared with controls. Cluster S2 contained both up- and down-regulated genes, and changes appear to be less significant than other clusters.

A summary of the top GO pathways deregulated in each cluster (S1–4, Fig 3C(ii) and table 2(i)), highlights key affected pathways that directly control male reproductive function e.g. regulation of sperm motility (S1), fusion of sperm to egg plasma membrane (S2) or prostate gland morphogenesis (S4). Differentially regulated genes within clusters 3 and 4 were also implicated in early developmental processes including embryonic morphogenesis and development, thereby inferring potential post-fertilisation function.

Moreover, as shown in Table 2(ii), genes that were significantly affected in *Brn-3b* KO testes were also strongly associated with GO pathways implicated in mitochondrial function (including mitochondrial protein assembly/translation) and electron transport chain (e.g. complex 1 and 4), which are critical for ATP production, sperm motility and function. Similarly, significant changes in GO pathways affecting sperm tail structure and function included axonemal dynein complex and microtubule motor activity (Table 2 (iii)). Other noteworthy pathways affected in *Brn-3b* KO testes were linked to cell signalling pathways, including androgen receptor pathway, estradiol/estrogen receptor associated processes, micro-RNA regulation, protein translation/ribosomal complexes and DNA remodelling. These results also confirmed that loss of *Brn-3b* in the testes affected multiple essential pathways that either directly or indirectly affect spermatogenesis and fertility and therefore indicate important roles for this regulator in controlling spermatogenesis and fertility in males.

### Enrichment profile identified using Differential Expression of Genes (DEG) analysis:

**Figure 4:**
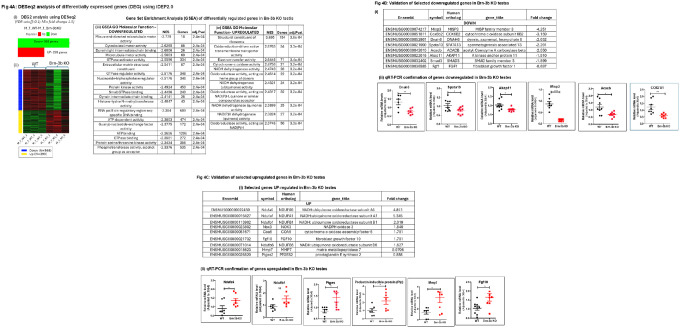
Differential Expression of Genes (DEG) analysis using iDEP platform: (A) Data from DEG analysis identifying the most significantly affected gene sets from top 2500 differentially expressed genes in WT control vs Brn-3b KO mutants, represented as (i) bar graph (ii) heat-map (FDR cutoff 0.1. Fold change cutoff 1.5). (iii-iv) Gene Set Enrichment Analysis (GSEA) of differentially regulated genes showing the most significant molecular functions that were down regulated (iii) or up regulated genes (iv) in mutant testes (p>0.005). (B) (i) Selected down regulated genes chosen for further validation studies were based on fold changeand functional effects (ii) qRT-PCR validation of selected genes from RNA sequencing data that were identified as down-regulated in Brn-3b KO mutants. (C) (i) Upregulated genes selected for further validation studies were based on fold change and functional effects (ii) qRT-PCR validation of selected genes from RNA sequencing data that were identified as upregulated in Brn-3b/POU4F2 KO mutants. * Indicates the statistical significance indicated by * (p>0.005), ns indicates non-significant effects.

To identify the top differentially expressed genes showing most significant changes in *Brn-3b* KO testes, DESeq2 analysis was next conducted as part of the DEG2 functionality in iDEP2.0^25,33^. Based on the selection criteria of p-value (<0.05) and fold change (≥1.5 fold), 568 genes were down-regulated and 293 genes were up-regulated in *Brn-3b* KO testes when compared with WT controls [Figure 4A(i) & (ii)]. Further in-depth analyses were conducted using Gene Set Enrichment Analysis (GSEA) combined with GO analysis of the top differentially regulated genes to identify key pathways and functions affected by loss of Brn-3b, with the normalisation enrichment score (NES) used to rank the top affected pathways. Fig 4A (iii) shows that genes down-regulated in *Brn-3b* KO testis affected molecular functions associated with sperm motility including microtubule motor activity, cytoskeletal motor activity and dynein complexes. Other noteworthy molecular functions affected by down regulated genes included RNA polymerase II activity and ATP-dependent activity, possibly associated with cellular respiration/electron transport chain, indicating potential downregulation of genes affecting such processes. On the other hand, the largest number of upregulated genes were associated with ribosomal function that may suggest deregulation of translation. However, consistent with previous analyses, most of the other upregulated pathways affected by loss of *Brn-3b* in the testis were linked to mitochondrial function and the electron transport chain, further implicating deregulation of bioenergetic function.

### Validation of selected target genes using qRT-PCR:

Based on these changes, a panel of highly differentially expressed genes were chosen for further validation studies [Fig. 4B(i) and 4C(i)] using cDNA synthesized from RNA isolated from independent *Brn-3b* KO and WT testes. Results of qRT-PCR confirmed significant changes in selected genes in *Brn-3b* KO testes. For example, genes showing significantly reduced expression in mutant testes included the dynein heavy-chain 6 (*Dnah6*), which encodes a key component of microtubule-associated motor protein complexes required for cilia motility^34^; *Spata13* or Adenomatous polyposis coli-stimulated guanine nucleotide exchange factor 2 (*Asef2*)^35^, and apical actin network regulator associated with cytoskeletal structure and function^36^ Akap11 (also known as Akap220) (Fig. 4B[ii]). Other genes down-regulated in mutant testes included *Misp3*, a member of the mitotic spindle protein (MISP) family, linked to cell cycle and chromatin remodelling^37^. Similarly, significant reduction in gene expression was seen in the *Cox7a1* gene encoding a subunit of the cytochrome C oxidase (complex IV), an essential component of the electron transport chain and Acetyl-CoA Carboxylase Beta gene, Acabc, which is involved in fatty acid uptake and oxidation by mitochondria^38^.

Conversely, validated genes that were increased in Brn-3b KO mutants are shown in Fig. 4C. RNA seq data highlighted increases in genes encoding NADH:ubiquinone oxidoreductase (NDUF) family (e.g. *Ndufa1* and *a6* and *Ndufb1* and *b6*), and validation studies confirmed significant increase in expression in *Ndufa6*, but non-significant increase for other NDUF genes (e.g. *Ndufb1*^39^). Such results suggest deregulation of multiple genes that are required for complex 1 and IV, which are essential for electron transport chain and ATP synthesis essential complexes in mitochondrial function and could therefore affect sperm function.

Similarly, genes associated with inflammatory processes were also upregulated in Brn-3b KO testes. This included increased prostaglandin E synthase (*Ptges* or *Pges*), which is associated with production of pro-inflammatory prostanoid, prostaglandin E_2_ (Pge2)^40^ and the secretory prolactin inducible glycoprotein, Pip which binds to the Fc fragment of immunoglobulin (IgG) and anti-sperm antibody^41^.

Loss of *Brn-3b* also caused increased expression of different proteases involved in ECM remodelling, including the matrix metalloproteinase proteases, *Mmp7*, linked to high sperm DNA fragmentation and apoptosis^42^. Increased expression of *Fgf10* in mutant testes suggests deregulation of signalling pathways^43^. Taken together, RNA sequencing and validation studies confirmed that loss of Brn-3b affected multiple groups of target genes that are implicated in testes development, spermatogenesis and sperm function.

### Analysis of *Brn-3b/POU4F2* expression in human testes using HISTA single cell testis atlas:

**Figure 5:**
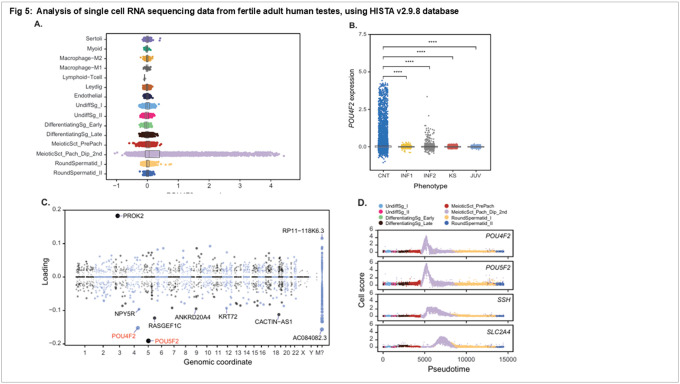
Analysis of single cell RNA sequencing data from fertile adult human testes, using HISTA v2.9.8 database. (A) Box plot showing *Brn-3b* (*POU4F2*)positive cell populations in control human testes were specifically enriched in pachytene spermatocytes. (B) Comparative analysis of *Brn-3b* mRNA-positive cell populations in human testes using single-cell RNA sequencing data from the HISTA database. The total testicular cell populations sampled from each phenotype are represented here. *Brn-3* bexpression is significantly higher in fertile adult males compared to men with diagnosed infertility or juvenile (JUV) testes. Infertile samples include INF1 (azoospermia), INF2 (ejaculatory dysfunction), and KS (Klinefelter syndrome) (C) Co-localization of *Brn-3b/POU4F2* with *POU5F2* within SDA component SDAV98, identified from single-cell RNA-seq analysis. *Brn-3b/POU4F2* and *POU5F2* emerged as strong outliers among genes with high negative loadings in this component. Gene clustering and cell scoring revealed overlapping expression of these genes in specific cell populations, supporting their coordinated activity during spermatogenesis. (D) Pseudotime trajectory analysis of *Brn-3b/POU4F2* (top) and two of its known targets, *SLC2A4* and *SHH* (bottom), reveals that target gene expression occurs primarily at later developmental or meiotic stages compared to *Brn-3b/POU4F2*, suggesting that *Brn-3b/POU4F2* expression precedes and may regulate their transcription during spermatogenesis.

*Brn-3b* expression has been reported in human testes^26,29–31^ but its relevance in male fertility is still not clear. Therefore, detailed analyses were next undertaken to analyse *Brn-3b* expression in human testes using the Human Infertility Single-cell Testes Atlas (HISTA) sequencing atlas containing data from 12 human donors (including testes biopsies from 6 normozoospermic adult human donors). This analysis revealed that *Brn-3b* mRNA is most highly expressed in pachytene spermatocytes (Fig 5A). Notably, when comparing *Brn-3b* mRNA expression patterns, we observed significantly higher expression levels in testis biopsies from normozoospermic controls compared to samples from infertile men, including individuals with non-obstructive azoospermia (INF1), a case of retrograde ejaculation (INF2), and patients with Klinefelter syndrome (KS). Moreover, expression levels were also significantly higher in controls compared to testis samples from juvenile males (Fig 5B). These expression patterns are consistent with the expected depletion or absence of meiotic and post-meiotic cells in these subjects.

Further investigation utilised the HISTA Structured Dimensionality Reduction (SDA) components to provide deeper insights into biological processes associated with *Brn-3b*. SDA is a method of soft clustering that identifies sets of co-expressed genes, which we call “components”, often corresponding to genes regulated by the same transcription factors and involved in the same biological processes^44,45^. *Brn-3b* was identified as a significant contributor to multiple SDA components, exhibiting a strong negative loading on SDAV141 (−0.404) and SDAV98 (−0.152), alongside positive loadings on SDAV21 (+0.191), SDAV71 (+0.096), SDAV74 (+0.094), and SDAV94 (+0.064). Gene Ontology (GO) enrichment analysis of these components elucidated their biological relevance in relation to *Brn-3b*. The most intriguing component involving *Brn-3b* is SDAV98 (Table 3). Strikingly, the top two negative gene loadings on this component are *Brn-3b (POU4F2)* and *POU5F2*, a POU family transcription factor and a paralog of *POU4F2*. Of the top 60 genes in this component, 39 are lncRNAs, and 4 are uncharacterized putative protein-coding genes (*C2orf78, C9orf57, C9orf163, C17orf96*). Most of the top 60 genes are poorly characterized, and several have been recently linked to human male infertility, including *C2orf78, POTEJ*, and *PROK2*.^31^ GO enrichments for this component all relate to plasma membrane cell-cell adhesion (Table 3).

For SDAV141, whose negative loading correlates with *Brn-3b/POU4F2* expression, the enriched GO terms from its negative loadings again indicate “cell-cell adhesion”, while positive (anticorrelated) gene loadings related to ‘Regulation of mitotic cell cycle phase transition,’ and “G2/M transition of mitotic cell cycle”, perhaps suggesting that *Brn-3b* expression may be coupled to a checkpoint or cell fate decision during meiosis. More broadly, GO term enrichment across the components revealed that *Brn-3b* is co-expressed with genes involved in late spermatogenic stages, encompassing functions such as ‘calcium-dependent cell-cell adhesion via plasma membrane adhesion,’ ‘male gamete generation,’ and ‘(single) fertilization.’

Given the molecular function of *Brn-3b* as a transcription factor, it is of interest to understand what genes might be activated by this protein in male germ cells. Pseudotime trajectory analysis of germ cells demonstrated that known *Brn-3b* targets, including *SLC2A4* (encoding GLUT4) and SHH (Sonic Hedgehog), were expressed in development steps just downstream of *Brn-3b* expression (Fig. 5D). Like *Brn-3b*, both genes have high loading on SDAV21, identifying a possible role for *Brn-3b* in the regulation of SDAV21 genes more broadly. GO categories represented in this component include “regulation of hormone secretion” and “sperm capacitation”. These patterns are consistent *Brn-3b* playing a causal role in the upregulation of wide range of downstream effectors during spermatogenesis.

### Identification of *POU4F2/Brn-3b* variants associated with male infertility:

To identify potentially deleterious genetic variants in the *Brn-3b* gene that could contribute to human male infertility, we analysed whole exome sequencing (WES) data from a large international cohort sequenced as part of the ongoing GEMINI project^31,46^.

**Figure 6:**
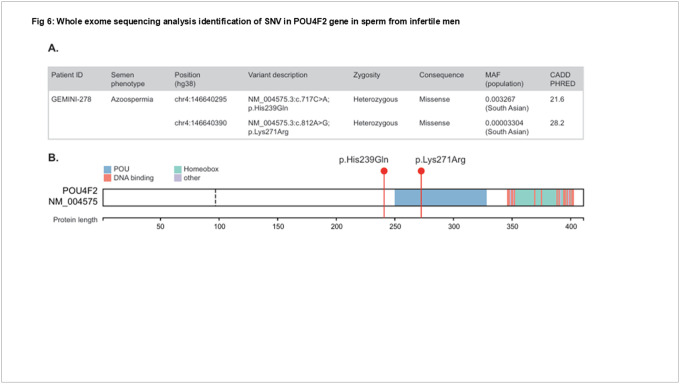
Clinical and molecular characterization of a *Brn-3b/POU4F2* variant in an azoospermia patient. (A) Table summarizing the patient ID, clinical phenotype, and variant information, including nucleotide and protein changes, zygosity, allele frequency and CADD score. (B) Schematic representation of the Brn-3b/POU4F2 protein showing the position of the variants in patient GEMINI-278 (red lines) relative to conserved domains.

After filtering, we identified one patient affected by non-obstructive azoospermia (NOA) carrying biallelic variants in *Brn-3b*. Specifically, this patient carried two deleterious missense variants with PHRED-scaled CADD scores greater than 20 (Fig 6). These were validated to be in trans; one of variant was located within the POU domain (Fig 6). Additionally, among nine other patients (six with NOA and three with extreme oligozoospermia), as well as seven normozoospermic controls, we observed heterozygous, rare, and predicted to be pathogenic *Brn-3b* variants (supplementary table 1). At present, one biallelic variants seem to be a strong contributor to the infertile phenotype while further studies will be necessary to determine the relevance of other *Brn-3b* SNVs in spermatogenesis and infertility.

## DISCUSSION

The escalating global prevalence of male infertility highlights an urgent need to elucidate the intricate molecular mechanisms governing viable sperm production^2,3^. The complex mechanisms associated with spermatogenesis mean that many fundamental processes have remained poorly understood, thereby creating significant challenges in identifying causal links in infertile men or developing effective therapeutic strategies. In this study, we present data obtained using a multifaceted approach to define novel and essential roles for the Brn-3b transcription factor in the regulation of spermatogenesis and male fertility. Our findings reveal a compelling association between compromised *Brn-3b* expression and infertility in both murine models and human subjects.

*Brn-3b* mRNA and protein expression was previously reported in mature spermatids in adult murine testes, with significantly lower levels in immature, juvenile testes^14^. Notably, Brn-3b expression was absent in infertile CREM mutants, which exhibit an arrest in mature spermatid development^14^. However, the direct impact of Brn-3b on testicular development and function has remained largely uncharacterized. Despite empirical observations of infertility in homozygous constitutive *Brn-3b* KO male mice, the precise mechanisms underlying these phenotypic changes were previously unknown.

Our comprehensive analysis of the constitutive *Brn-3b* KO model provides critical insights into these underlying cellular and molecular alterations. Detailed analysis of constitutive *Brn-3b* KO mice reveal pronounced structural and morphological abnormalities in developing spermatids, which correlated with low sperm counts as well as functional changes including reduced sperm motility. TEM images further highlighted significant ultrastructural abnormalities in *Brn-3b* KO sperm, including disrupted acrosomal membranes, mitochondrial disorganisation and fragmentation in the mid-piece, as well as abnormal changes in the anterior portion of ODFs and fibrous sheath of the sperm tail. Such abnormalities in the mutant sperm are consistent with known defects in infertile individuals and collectively indicate bioenergetic and functional deficiencies^46–48^.

Transcriptomic sequencing of *Brn-3b* KO mouse testes, compared to WT controls, also provided molecular insights into the mechanisms underlying identified phenotypic changes. For instance, functional annotation and pathway analyses showed that loss of *Brn-3b* significantly affected key gene ontology pathways associated with male gamete generation and spermatogenesis, respiratory chain and mitochondrial function or sperm motility including microtubule motor activity. Furthermore, deregulation of pathways involved in sperm-egg plasma membrane fusion aligns with the observed acrosomal defects, indicating a molecular basis for this functional impairment. The cap-like acrosome organelle at the sperm head contains hydrolytic enzymes, which are essential for penetrating the outer layers (zona pellucida and corona radiata) of the egg during fertilization. Disrupted acrosomal membranes, as observed in our TEM data, can lead to premature or ineffective acrosome reactions, impairing sperm-egg binding and preventing successful fertilization^8^. This is a well-established cause of male infertility, exemplified by conditions like globozoospermia where the acrosome is absent or severely malformed^48^.

Furthermore, spermatogenesis is highly dependent on normal mitochondrial function including energy production for essential processes including sperm motility, capacitation and acrosome reaction. This is reflected in the highly ordered mitochondrial sheath within the mid-piece of the mature spermatids^49^ and documented evidence showing that mitochondrial dysfunction is closely associated with compromised sperm motility and fertilisation^47^. Therefore, RNA sequencing data revealing significant disruption in mitochondrial pathways in *Brn-3b* KO testes are highly significant. It is also consistent with the pronounced ultrastructural mitochondrial abnormalities, notably fragmentation and disorganisation in mitochondria around the mid-piece of *Brn-3b* KO spermatids and correlates with reduced sperm motility in *Brn-3b* KO mutants.

Importantly, pathway analysis of deregulated processes in *Brn-3b* KO mouse testes revealed disruption of mitochondrial pathways, notably associated with oxidative phosphorylation, ATP synthesis and respiratory chain activity within the inner mitochondrial membrane. Such changes are noteworthy because of dependence on normal mitochondria function during spermatogenesis but also in regulating sperm function. Furthermore, the relative abundance of mitochondria especially in the mid-piece of the spermatozoa also highlights its central role in normal sperm function^9^. Thus, in addition to the ATP production via the electron transport chain to support spermatogenesis and sperm function including motility, mitochondrial function is also implicated in the production of steroid hormones in the testes, control of cell proliferation, sperm-egg interaction via regulated production of reactive oxygen species production (ROS) but also in controlling apoptosis^9,49^. Based on such diverse function, normal mitochondrial function is essential for spermatogenesis, sperm function and fertility.

In this regard, qPCR validation studies showing significantly increased expression of including increased expression in NDUF genes such as *Ndufa6* but reduction of *Cox7a1* in independent *Brn-3b* KO testes confirms disruption of mitochondrial genes associated with key complexes 1 and IV, which are critical for the electron transport chain and ATP synthesis.

For example, *NDUF* (NADH:ubiquinone oxidoreductase) genes encode critical components of the proton translocation module within mitochondrial Complex I,^49^, which is essential for generating the proton gradient that drives ATP synthesis. Overexpression of key subunits such as *Ndufa6*, while not a catalytic subunit, can disrupt the stoichiometry and functional integrity of Complex I and impair the efficiency of electron transfer and disrupt the electron transport chain. Similarly, downregulation of Cox7a1, which encodes a subunit of cytochrome c oxidase (COX; Complex IV), may compromise completion of the electron transport chain. As Complex IV functions as the terminal enzyme responsible for transferring electrons to molecular oxygen, its dysfunction can severely impair oxidative phosphorylation, leading to reduced ATP production and elevated reactive oxygen species (ROS)^50,51^.

Such disruption of key complexes within the mitochondrial electron transport chain in *Brn-3b* KO testes is therefore likely to contribute to diminished ATP synthesis, which in turn affects energy-dependent processes such as flagellar movement essential for sperm motility, as well as capacitation and the acrosome reaction which are both critical for sperm-egg fusion and successful fertilisation. These functional impairments align with the cellular pathways disrupted in *Brn-3b* KO testes.

Moreover, perturbation of the electron transport chain and increased electron leakage are strongly associated with abnormally elevated ROS production and resultant oxidative stress, commonly associated with sperm damage and male infertility^49^. This is consistent with the ultrastructural abnormalities revealed by TEM imaging of Brn-3b KO sperm, including acrosomal and flagellar damage and mitochondrial fragmentation, associated with sperm damage and infertility^52 39^. While the precise mechanisms by which *Brn-3b* regulates mitochondrial processes in sperm remain to be fully elucidated, current findings suggest that impaired mitochondrial function in *Brn-3b* KO testes compromises the bioenergetic capacity of spermatozoa required for motility and fertilisation. Such dysfunctions likely contribute to infertility phenotypes such as asthenozoospermia (reduced sperm motility) and other forms of male infertility^47,49^.

Deregulation of genes associated with structural proteins also corroborate the ultrastructural defects observed in the fibrous sheath and outer dense fibres of *Brn-3b* KO sperm flagellum. The validated downregulation of structural genes encoding dynein heavy-chain motor proteins such as *Dnah6* in *Brn-3b* KO mutants are consistent with impaired motility observed in *Brn-3b* KO sperm. Such structural proteins are essential for cilia motility and synchronised flagellar beating necessary for progressive motility and disruption in expression or function can contribute to asthenozoospermia^34,53,54^. Similarly, confirmed reduction of other spermatogenesis-associated genes such as *Spata13*, linked to cell migration and adhesion, may potentially affect sperm motility and fusion capability^55^ while down-regulation of *Misp3* (involved in spindle formation and cell cycle progression)^37^ suggest broader disruptions in germ cell development. The combination of structural disruption in sperm tail accessory structures involved in cytoskeletal architecture and motility strongly implicates Brn-3b in preserving the structural and functional integrity of the sperm tail.

Interestingly, *Brn-3b* KO testes exhibit upregulation of inflammatory mediators including prostaglandin E synthase (*PTGES* or *PGES*), which catalyses the synthesis of pro-inflammatory prostanoid, PGE2^40^. Additionally, elevated expression of the secretory prolactin inducible glycoprotein, *Pip*, in Brn-3b KO testes is noteworthy because of its ability to bind to Fc fragment of IgG and anti-sperm antibody, suggesting a potential role in immunological dysregulation^41^. Given the established link between chronic inflammation and male infertility, up-regulation of such genes may represent either compensatory responses to defective spermatogenesis or contribute directly to unfavourable microenvironment for developing germ cells, thereby contributing to infertility^52,56^. Furthermore, loss of *Brn-3b* also affected expression of proteases involved in ECM remodelling, with confirmed increases in matrix metalloproteinase proteases, *Mmp7*, which is implicated in sperm DNA fragmentation and apoptosis^42^ while increased expression of *Fgf10* in mutant testes suggests deregulation of signalling pathways^43^. These changes are likely due to apoptosis of malformed spermatids and results in lower number of elongated spermatids as shown in Fig. 1.

Comprehensive analyses of *Brn-3b* KO testes have provided mechanistic insights linking loss of *Brn-3b* with defective spermatogenesis, characterised by abnormalities in sperm morphology, bioenergetic capacity and functional integrity. Although whole genome sequencing of testes from WT and Brn-3b KO mice must be interpreted with caution based on issue heterogeneity and potential bias in cellular composition in mutant testes, the observed molecular changes are consistent with infertility phenotype seen in male homozygous *Brn-3b* KO mutants. These findings reinforce the role of Brn-3b in maintaining reproductive function and provide further evidence of the morphological, genetic and functional disruptions that result from loss of Brn-3b.

Since Brn-3b is highly conserved between humans and mice, sharing more than 98% amino acid homology, our data from the Brn-3b KO mutant mice could have strong translational relevance for understanding the mechanisms of spermatogenesis in humans and understanding the mechanisms and basis of male infertility in the clinical setting. Multiple reports of Brn-3b/POU4F2 expression in human testis^26–29^ and identification of Brn-3b/POU4F2 as a gene of interest in male fertility^29–31^ is also supported our data from analysis of single-cell RNA sequencing data from the HISTA database, which show strong *POU4F2* (*Brn-3b*) expression in pachytene spermatocytes from fertile men. These findings are consistent with mouse studies that demonstrate Brn-3b protein expression in both spermatocytes and spermatids from wild-type (WT) mice and its absence in CREM knockout (KO) mutants, which lack this cell population^14^.

In line with the infertility observed in *Brn-3b* knockout mice, scRNA-seq data from human testes also reveal significantly reduced populations of *POU4F2* (*Brn-3b*)-expressing cells in infertile men diagnosed with azoospermia and Klinefelter syndrome. These reduced levels are comparable to the low *POU4F2* (*Brn-3b*) expression seen in immature juvenile testes, which can be primarily attributed to the lack or substantial reduction of spermatocytes and spermatids in these conditions compared to fertile controls. Furthermore, gene ontology (GO) term enrichment analysis demonstrates that *POU4F2* (*Brn-3b*) expression significantly correlates with other genes crucial for late spermatogenic stages and fertilization. Moreover, its expression dynamics align with existing literature, as pseudotime analysis confirms that known POU4F2 targets, such as *SLC2A4* (*GLUT4*) and *SHH*, are expressed immediately subsequent to *POU4F2* (*Brn-3b*) expression along the spermatogenic trajectory.

Taking all this evidence together, it strongly suggests that *POU4F2* may also play a crucial role in human male infertility. To investigate this, we performed a targeted search for rare and potentially pathogenic variants in a cohort of 1,474 men with idiopathic azoospermia/ oligozoospermia and 633 normozoospermic controls, sequenced within the GEMINI cohort. This analysis revealed a potential genetic cause of human male infertility in a patient with Non-Obstructive Azoospermia, who carries two rare missense variants in *POU4F2* (*Brn-3b*). One variant was inherited from the father and the other from the mother, both exhibiting high CADD scores, indicating their likely deleteriousness. Although the functional consequences of these variants remain to be investigated, these findings, combined with data from Brn-3b knockout (KO) mutant mice, may offer important leads for understanding the genetic basis of male infertility in future studies.

In conclusion, this study provides strong evidence supporting a critical role for Brn-3b in spermatogenesis and male fertility. Compelling evidence from Brn-3b knockout (KO) mouse models has highlighted the essential roles of Brn-3b in controlling spermatogenesis, sperm development, and function. The observed infertility in Brn-3b knockout mice, coupled with single-cell RNA sequencing (scRNA-seq) data revealing significantly reduced populations of *POU4F2* (*Brn-3b*)-expressing cells in infertile men with azoospermia and Klinefelter syndrome, highlights its importance in human spermatogenesis. Furthermore, the identification of rare and potentially pathogenic *POU4F2* (*Brn-3b*) missense variants in an infertile man with Non-Obstructive Azoospermia provides compelling human genetic evidence supporting its etiologic role. These combined lines of evidence underscore the urgent need for future investigations into the functional consequences of *POU4F2* (*Brn-3b*) variants in the context of human male infertility. Ultimately, a deeper understanding of the genetic and molecular contributions of *POU4F2* (*Brn-3b*) could pave the way for novel diagnostic, prognostic, or therapeutic strategies to address male infertility and improve reproductive outcomes for affected couples.

## MATERIALS AND METHODS

### Materials:

General laboratory reagents: Merck (Nottingham, UK) and Sigma (Dorset, UK), unless otherwise stated.

### qRT-PCR Primer Sequences:

Brn-3b F - 5’ GAGAGAGCGCTCACAATTCC 3’

Brn3b R - 5’ ATGGTGGTGGTGGCTCTTAC 3’

36b4 F - 5’ AGATGCAGCAGATCCGCAT 3’

36b4 R - 5’ GTTCTTGCCCATCAGCACC 3’

Gapdh F - 5’ CTTCATTGACCTCAACTAC 3’

Gapdh R - 5’ AGTGATGGCATGGACTGTG 3’

Spata13 F - 5’ GGCTTTGGAGCTTCGGTGGA 3’

Spata13 R - 5’ TCCTGGGCTGTTGTCATGTTC 3’

Akap11 F - 5’ CCACACACGGGTAGGGAAAA 3’

Akap11 R - 5’ TTGAGTACGCCGCTTCCAAT 3’

Misp3 F - 5’ GAAGATGCAACGGGACATCG 3’

Misp3 R - 5’ AAGCGCTTGAGTTCGTCCAG 3’

Acacb F - 5’ GGGAATGCAAGGCCAAAGTG 3’

Acacb R - 5’ ACCTTACTGTTGGTGAGCGG 3’

Cox7a1 F - 5’ GCTGCTGAGGACGCAAAATG 3’

Cox7a1 R - 5’ TTAGGCATCTGGGTTGTGGG 3’

### Methods:

#### Mouse studies: Testes dissection and processing

All animal procedures, including breeding and maintenance of genetically altered mice, were carried out in accordance with the UK Animals (Scientific Procedures) Act 1986 and approved by the institutional Animal Welfare and Ethical Review Body. Studies were undertaken using Brn3b KO mice and age/sex matched WT littermate controls obtained by crossing inbred Brn-3b heterozygote males and females^57^. For sample preparation, experimental mice were culled by rising concentration of CO_2_, prior to dissection of the epididymis for isolating sperm or the testes, that was either snap frozen (in liquid nitrogen) or fixed in 4% paraformaldehyde for downstream processing and analyses using molecular or histological studies.

##### Paraffin embedded whole testes and histological analysis of testes sections

For in-depth histological and immunocytochemistry studies, the testes and epididymis dissected from approximately 3-month-old WT and *Brn-3b* KO mice were fixed in 4% paraformaldehyde overnight, washed in PBS and embedded in paraffin wax, using the Tissue-Tek tissue processer (Sakura Finetek). Paraffin-embedded blocks were cut into 5–7μm sections using a microtome. Haematoxylin and eosin staining was done using the auto stainer and samples were imaged using the Nanozoomer 2.0 (Hamamatsu) imager.

Quantification of seminiferous tubule diameter was undertake using ImageJ (FiJi) software. The average measurement of multiple tubules from different sections of the same sample were combined with values derived from multiple independent WT and Brn-3b KO testes were used to calculate average tubule sizes.

###### Isolated sperm and germ-cell samples:

For analysis of isolated mature sperm, the cauda of the epididymis was dissected away from the rest of the reproductive tract and put into phosphate buffered saline (PBS). Cauda was snipped and incubated in PBS at 37°C for 15 minutes to allow sperm to swim out. This solution was then mixed gently and transferred to fresh tube, leaving remaining tissue behind. Sperm count was undertaken using an aliquot of isolated sperm cell solution (total volume of 2 cauda epididymis 1ml) that was diluted 1:10, then counted with a haemocytometer. Fresh cells were also used for live cell imaging (below) and for preparing sperm smears on slides for histological and immunofluorescent staining. Germ cells were also isolated by decapsulating whole testes to release germ cells from the seminiferous tubules. Tubule pieces were transferred to a solution of 100mM sucrose and then cut to release germ cells. The solution was pipetted up and down several times until cloudy to ensure release of germ cells.

##### Live cell imaging

Live cell imaging was used to analyse changes in morphology and motility in freshly isolated sperm samples. For motility studies, mature sperm samples were prepared as described above and 100μl sperm solution was transferred to a 24 well cell culture plate for time-lapse images using the Leica DMi8 inverted microscope (at 20x magnification). Videos were taken at 3–5 areas of each sperm sample, using the maximum frames-per-second rate for 30–90 seconds. Files were exported as .lif files for further analysis, using FiJi /ImageJ software.

For image processing and data analysis of motile and nonmotile sperm, DIC captured movies of sperm were imported into Wolfram Mathematica 13 (Champaign, Il). Each frame was background subtracted with a 51 pixel radius Gaussian filter. The absolute magnitude of this was taken and brightness auto-adjusted. This created images where the whole sperm head was clearly visible. Each frame was then colour coded from red, for the earliest time points, through a rainbow LUT, to purple for the later time points. Each frame was then overlaid in a manner that showed sperm motility, or lack thereof. Motile sperm appear as rainbow-coloured ‘comet-tails’, whereas immotile sperm appear as white blobs. These could be easily segmented by identifying white pixels and non-white pixels allowing easy visualisation of motile and non-motile populations.

#### RNA extraction

RNA was extracted from snap frozen mouse testes using the RNeasy Plus Mini Kit (Qiagen, Manchester, UK) following manufacturers’ protocol. For tissue disruption, mouse testes snap frozen in liquid nitrogen were ground to a fine powder using a pestle and mortar, then transferred to an Eppendorf tube for further processing. For 30mg of tissue 800μl of RLT buffer with β-mercaptoethanol was added to a sample before homogenising the samples for 30–60 seconds, (using handheld rotor stator homogeniser). Following centrifugation (13,000 rpm for 2 mins) to remove cell debris, genomic DNA was eliminated from the supernatant using gDNA eliminator columns. RNA was extracted from the supernatant using Qiagen RNeasyPlus Mini kit (Qiagen, Manchester, UK). Purified RNA quantified by nanodrop was used for cDNA synthesis.

### Transcriptome analysis:

RNA sequencing was undertaken by Novogene (Cambridge, UK) using total RNA prepared from Brn-3b KO testis and age-matched wild-type controls (>3/set). Briefly this included quality control (QC) of RNA samples, mRNA library preparation (poly A enrichment) and sequencing using NovaSeq X -PE 150(Illumina, identifier A01426; 15 G raw data per sample). Raw data was stored in FASTQ (fq) format files, which contain sequences of reads and corresponding base quality. Sequence alignment was done using HISAT2 followed by mapping to referencing genome. De-multiplexed data converted to Fastq files were subject to standard analysis with reference (WBI-quantification). This data provided by the company including information on gene expression levels was subject to further in-depth analysis for differential gene expression and functional changes (DESeq2, edgeR; GSEA analysis), enrichment analysis (cluster profile hierarchical clustering and Gene Ontology (GO) enrichment pathway analysis), and statistical analysis.

Additional bioinformatic analysis of RNA sequencing data from Brn-3b KO and WT testes were also carried out using the BigOmics Analytics^32^ and iDEP2.0^33^ platforms, to identify pathways affected upon loss of *Brn-3b* in the testis. For these studies, preprocessing was used to filter genes, based on variance, expression of samples and missing values and normalised counts were then used for in-depth analysis. Clustering module using principal component analysis (PCA) was used to analyse for variance between samples of similar genotype and the differential expression analysis was used to generate heat maps of samples based on fold changes and adjusted p-values. For network analysis, the differentially regulated genes were assembled into four independent clusters based on functional differences associated with the gene sets. Gene Set Enrichment Analysis (GSEA) and DESeq2 analysis of Differential Expression of Genes (DEG2) methods were used to identify differentially regulated gene sets, features or pathways in *Brn-3b* KO vs WT control testes [minimum fold change of 1.5 and false discovery rate (FDR) cut off of 0.1].

### Gene validation: cDNA synthesis and quantitative polymerase chain reaction (qRT PCR):

Selected differentially regulated genes from RNA sequencing analysis were validated using RNA extracted from independent snap-frozen testes taken from *Brn-3b* KO and WT mice. RNA extraction was performed as described above and contaminating genomic DNA was eliminated prior to cDNA synthesis, using RNAse-free DNAse1 (Promega, Southampton, UK). 1mg of total RNA from each sample was used for cDNA synthesis, using Superscript^™^ II Reverse Transcriptase (Invitrogen, UK), according to the manufacturer’s protocol.

Validation studies were conducted to confirm changes in selected genes from RNA sequencing using qRT-PCR carried out with the Eppendorf Mastercycler using SYBR Green chemistry. Gene specific primers were designed for each target gene and used to amplify cDNA from WT and KO testes. Housekeeping genes, 36B4 and/or GAPDH, were used for normalization between samples. Reference samples were included in each experiment and used to calculate fold changes in relation to control samples using DDCT method. Statistical analysis was undertaken using results from multiple experiments and student’s t-test or ANOVA to show significance, *p≤0.05.

### Transmission electron microscopy (TEM):

Detailed ultrastructural analysis using TEM was undertaken using testes from WT and *Brn-3b* KO male mice (approximately 12 weeks old) or from sperm samples prepared from similar aged animals. For testes preparations, the seminiferous tubules were released by decapsulation prior to fixation and processing for TEM using standard protocol (Dr E. Slavik-Smith; UCL Division of Biosciences Electron Microscopy Facility). Sperm samples were prepared by separating the cauda of the epididymis followed by incubation in PBS at 37°C to facilitate sperm release. Following centrifugation, sperm pellets were fixed, processed and embedded for TEM imaging Briefly, samples for TEM were fixed (2.5% glutaraldehyde in 0.1M sodium cacodylate buffer; overnight at 4°C), washed, post-fixed in 1% osmium tetroxide buffer (1–2 hr at 4oC), followed by dehydration and embedding in Araldite resin. Ultrathin sections (50–70 nm) from each sample were collected on copper grids, counterstained, then imaged using the Jeol 2100 (200 kV) Transmission Electron Microscope (Prof J. Burden; UCL LMCB).

### Human studies integrating single-cell expression and exome variant data:

Transcriptomic data from human testis samples were analyzed using established single cell RNA sequencing datasets generated from high quality testicular cells isolated from biopsies of 12 human donors, as part of the Human Infertility Single-cell Testis Atlas (HISTA) and stored at https://conradlab.shinyapps.io/HISTA/^44^.

To identify POU4F2-related variants potentially associated with male infertility, we analysed whole exome sequencing (WES) data from GEnetics of Male INfertility Initiative (GEMINI)^31,46^, which includes 1,474 unrelated men diagnosed with spermatogenic failure and 633 fertile controls. We performed a targeted search for rare single nucleotide and frameshift variants in *POU4F2* (the human ortholog of *Brn-3b/POU4F2*), defined as those with a maximum allele frequency <0.01 in gnomAD (https://gnomad.broadinstitute.org/) and a Combined Annotation Dependent Depletion (CADD)^58^ score >20, indicative of predicted pathogenicity.

## RESULTS

### Loss of Brn-3b affects seminiferous tubule diameter and spermatid differentiation:

Based on *Brn-3b* expression in mature spermatids of adult mouse testes(14) and infertility in constitutive male Brn-*3b KO* mice, this model was used to investigate the impact of *Brn-3b* loss on testis morphology, spermatogenesis, and sperm structure.

To investigate whether loss of Brn-3b induces morphological or histological changes in the testes, we analysed multiple H&E-stained sections from independent wild-type (WT) and Brn-3b knockout (KO) mouse testes at varying magnifications. Figure 1A[i] presents representative cross-sectional images of stage V–VIII seminiferous tubules from WT testis sections. At higher magnification, WT tubules exhibit a well-organized arrangement of germ cell populations with round, immature spermatogonia (Sg) located at the periphery of the tubules, while spermatocytes (Sc) and mature spermatids are progressively positioned toward the lumen. Compact, elongated heads of spermatozoa (Sz) are visible around the lumen (arrow), with long tails projecting into the lumen (Fig. 1A[i], 40× magnification). In contrast, Brn-3b KO testis sections show that despite a comparable distribution of immature spermatogonia and spermatocytes, there is a marked reduction in the number of elongated spermatids near the lumen (Fig. 1A[ii], arrows) compared to WT controls.

Measurement of average seminiferous tubule diameter across independent experimental groups revealed that *Brn-3b KO* mutants exhibited significantly reduced tubule diameter compared to WT controls (Fig. 1A[iii]). This reduction may indicate a direct structural role for Brn-3b in maintaining seminiferous tubule architecture, or alternatively, reflect a secondary consequence of decreased numbers of elongating spermatids within the tubules.

### Low sperm quality and motility in Brn-3b mutants:

To determine whether loss of Brn-3b affects sperm structure and function, sperm samples were collected from the dissected epididymides of age-matched wild-type (WT) and Brn-3b knockout (KO) mice. Sperm cell numbers in each sample were quantified using either a haemocytometer or an automated cell counter, and data from independent WT and Brn-3b KO mice (n = 8 per group) were plotted (see Methods). As shown in Fig. 1B[i], sperm counts from Brn-3b KO mice were significantly lower than those from WT controls (unpaired t-test, p < 0.01).

Time-lapse contrast-enhanced imaging of isolated live sperm (Fig. 1B[ii]) further confirmed the reduced sperm numbers in Brn-3b knockout (KO) mutants. Additional image processing was performed to distinguish motile from non-motile sperm cells, using colour enhancement to highlight positional changes in highly motile cells within each sample. Representative images in Fig. 1B[iii], which highlight motile sperm cells, show a markedly higher number of motile cells in wild-type (WT) controls (top panel) compared to Brn-3b KO samples (lower panel). Non-motile cells, shown in Fig. 1B[iv], also demonstrate reduced cell numbers in Brn-3b KO sperm samples, consistent with reduced sperm count.

These findings confirm that Brn-3b KO male mice produce fewer mature sperm cells in the epididymis, with a substantial reduction in motile sperm. This defect is highly likely to contribute to infertility observed in the mutant mice.

### Ultrastructural changes in elongated spermatids from Brn-3b KO mutants show disruption of sperm tail accessory structures and the acrosome:

TEM was used to analyse for ultrastructural changes in *Brn-3b* KO sperm, when compared with age-matched WT controls. Representative images in Fig. 2, show that the most significant changes observed in *Brn-3b* KO spermatids were linked to the acrosome, midpiece and fibrous sheath. For instance, while WT spermatids displayed a compact head with condensed nucleus surrounded by a well-defined acrosome (Fig. 2A; top panels), comparable spermatids from *Brn-3b* KO displayed marked acrosomal changes, with membrane detachment and acrosomal fragmentation into multi-vesiculated and disrupted structures (Fig. 2A; lower panels).

Similarly, Fig. 2B shows changes in the mid-piece, with WT spermatids displaying a well-defined mitochondrial sheath comprising intact, rounded, and well-organized mitochondria, which was contiguous with the well-organised principal piece of the flagellum. In contrast, the mitochondrial sheath in the mid-piece of *Brn-3b* KO spermatids appeared less organised with smaller mitochondrial components. Moreover, the adjoining fibrous sheath of the principal piece also appears disrupted. The differences in mitochondrial organisation around the mid-piece are also highlighted in Fig. 2C where the mitochondrial content of the mid-piece in WT spermatids displayed highly regular mitochondrial structures with defined membranes around the mitochondrial sheath while mid-piece from *Brn-3b* KO sperm, displayed smaller, more irregular mitochondrial structure with some evidence of fragmentation in mitochondrial content (lower panels). Furthermore, the principal piece of the flagella of sperm from *Brn-3b* KO mice also exhibited fibrous sheath dysplasia, with disruption and structural disorganization of the fibrous sheath and the outer dense fibres (ODF) when compared with the contiguous and intact fibrous sheaths and ODFs seen in control spermatids (Fig. 2D).

These ultrastructural abnormalities in *Brn-3b* KO sperm, which include acrosomal defects, disorganization of the mitochondrial and fibrous sheath aligns with observation of infertility in *Brn-3b* KO male mice and may also explain our observation of reduced sperm count and poor motility in *Brn-3b* KO sperm.

### Deregulation of genes and pathways linked to male gamete generation, spermatogenesis, motility and bioenergetics in Brn-3b KO testes.

As a transcription factor, Brn-3b protein exerts diverse cellular effects by regulating the expression of specific target genes, in a highly tissue dependent manner. Therefore, infertility in male *Brn-3b* KO mice and the associated histological, functional and ultrastructural abnormalities in mutant sperm are likely to result from in changes in critical gene pathways that could provide insight into the molecular mechanisms for controlling spermatogenesis. As such, RNA sequencing analysis was next undertaken to identify genes that are differentially in testes from *Brn-3b* mutants when compared with WT controls.

RNA extracted from independent testes of *Brn-3b* KO mice and WT controls was used for high throughput RNA sequencing and bioinformatics analyses. Figure 3A shows (i) the volcano plot and (ii) heat map of differentially expressed genes (p value < 0.05), with 2242 upregulated genes and 2653 down regulated genes in KO testes when compared with WT controls. Gene Ontology (GO) analyses of differentially regulated genes highlight the top biological processes (BP), cellular components (CC) and molecular functions (MF) affected in *Brn-3b* KO testes when compared with WT controls (Fig. 3B [i]). The number of genes associated with selected pathways are shown in Fig. 3B[ii]. The top biological processes affected by loss of Brn-3b in the testes were linked to male gamete generation (179 genes) and spermatogenesis (170 genes) highlighting key roles for Brn-3b in regulating such processes. The hierarchical network of enriched biological processes shown in the directed acyclic graph (DAG) (Supplementary Fig. 1) shows that several nodes affected in mutant testes converge on biological processes associated with sexual reproduction, specifically male gamete generation (GO.004823276) and spermatogenesis (GO.0007283). The top genes associated with these pathways (Table 1) include families of proteins with known functions in spermatogenesis e.g. SPATA; DNAH and AKAP proteins.

Other biological processes affected in *Brn-3b* KO testes included ribonucleic complex biogenesis and assembly (152 genes), microtubule-based movement (106 genes) and mitochondrial respiratory chain complex assembly (44 genes). Analysis of the cellular components affected by loss of Brn-3b in testes, shows that in line with the biological processes, differentially regulated genes were associated with either the ribosomal complex or the mitochondrial complexes, including the respiratory chain suggesting key functions in protein translation and metabolic processes linked to oxidative phosphorylation and bioenergetics. These findings were also consistent with molecular functions, with differentially regulated genes involved in GTPase binding and activity and ATPase-coupled activities as well as cell motility (dynein chain binding and microtubule/motor tubule activity). This data suggests that loss of *Brn-3b* in the testes profoundly affects spermatogenesis and sperm function but also caused disruption in mitochondrial bioenergetics and motility related activities.

The effects of loss of *Brn-3b* on gene ontology pathways was also confirmed by undertaking additional bioinformatics analyses using the BigOmics Analytics platforms(33), which classifies differentially expressed genes into clusters based on top-ranked features (by correlation) and subsequent functional annotation of gene modules in each of the four clusters. The heat map of top-ranked differentially regulated genes, shown in Fig. 3C(i) highlights the overall changes in each cluster, with differentially expressed genes in clusters S1 and S3 being primarily down-regulated in *Brn-3b* KO testis while cluster S4 contained genes that were mainly upregulated in mutants when compared with controls. Cluster S2 contained both up- and down-regulated genes, and changes appear less significant than other clusters.

A summary of the top GO pathways deregulated in each cluster (S1–4, Fig. 3C(ii) and table 2(i)), highlights key affected pathways that directly control male reproductive function e.g. regulation of sperm motility (S1), fusion of sperm to egg plasma membrane (S2) or prostate gland morphogenesis (S4). Differentially regulated genes within clusters 3 and 4 were also implicated in early developmental processes including embryonic morphogenesis and development, thereby inferring potential post-fertilisation function.

Moreover, as shown in Table 2(ii), genes that were significantly affected in *Brn-3b* KO testes were also strongly associated with GO pathways implicated in mitochondrial function (including mitochondrial protein assembly/translation) and electron transport chain (e.g. complex 1 and 4), which are critical for ATP production, sperm motility and function. Similarly, significant changes in GO pathways affecting sperm tail structure and function included axonemal dynein complex and microtubule motor activity. Other noteworthy pathways affected in *Brn-3b* KO testes were linked to cell signalling pathways, including androgen receptor pathway, estradiol/estrogen receptor associated processes, micro-RNA regulation, protein translation/ribosomal complexes and DNA remodelling. These results also confirmed that loss of *Brn-3b* in the testes affected multiple essential pathways that either directly or indirectly affect spermatogenesis and fertility and therefore indicate important roles for this regulator in controlling spermatogenesis and fertility in males.

### Enrichment profile using Differential Expression of Genes (DEG) analysis:

To identify the top differentially expressed genes showing most significantly changes in *Brn-3b* KO testes, DESeq2 analysis was next conducted as part of the DEG2 functionality in iDEP2.0 (25, 34). Based on the selection criteria of p-value (< 0.05) and fold change (≥ 1.5 fold), 568 genes were down-regulated and 293 genes were up-regulated in *Brn-3b* KO testes when compared with WT controls [Figure 4A(i) & (ii)]. Further in-depth analyses were conducted using Gene Set Enrichment Analysis (GSEA) combined with GO analysis of the top differentially regulated genes to identify key pathways and functions affected by loss of Brn-3b, with the normalisation enrichment score (NES) used to rank the top affected pathways. Figure 4A (iii) shows that genes down-regulated in *Brn-3b* KO testis affected molecular functions associated with sperm motility including microtubule motor activity, cytoskeletal motor activity and dynein complexes. Other noteworthy molecular functions affected by down regulated genes included RNA polymerase II activity and ATP-dependent activity, possibly associated with cellular respiration/electron transport chain, indicating potential downregulation of genes affecting such processes. On the other hand, the largest number of upregulated genes were associated with ribosomal function that may suggest deregulation of translation. However, consistent with previous analyses, most of the other upregulated pathways affected by loss of *Brn-3b* in the testis were linked to mitochondrial function and the electron transport chain, further implicating deregulation of bioenergetic function.

### Validation of selected target genes using qRT-PCR:

Based on these changes, a panel of highly differentially expressed genes were chosen for further validation studies [Fig. 4B(i) and 4C(i)] using cDNA synthesized from RNA isolated from independent *Brn-3b* KO and WT testes. Results of qRT-PCR confirmed significant changes in selected genes in *Brn-3b* KO testes. For example, genes showing significantly reduced expression in mutant testes included the dynein heavy-chain 6 (*Dnah6*), which encodes a key component of microtubule-associated motor protein complexes required for cilia motility(38); *Spata13* or Adenomatous polyposis coli-stimulated guanine nucleotide exchange factor 2 (*Asef2*)(39), and apical actin network regulator associated with cytoskeletal structure and function(40)Akap11 (also known as Akap220) (Fig. 4B[ii]). Other genes downregulated in mutant testes included *Misp3*, a member of the mitotic spindle protein (MISP) family, linked to cell cycle and chromatin remodelling(41). Similarly, significant reduction in gene expression was seen in the *Cox7a1* gene encoding a subunit of the cytochrome C oxidase (complex IV), an essential component of the electron transport chain and Acetyl-CoA Carboxylase Beta gene, *Acabc*, which is involved in fatty acid uptake and oxidation by mitochondria(42).

Conversely, validated genes that were increased in Brn-3b KO mutants are shown in Fig. 4C. RNA seq data highlighted increases in genes encoding NADH:ubiquinone oxidoreductase (NDUF) family (e.g. *Ndufa1* and *a6* and *Ndufb1* and *b6*), and validation studies confirmed significant increase in expression in *Ndufa6*, but non-significant increase for other NDUF genes (e.g. *Ndufb1*(43)). Such results suggest deregulation of multiple genes that are required for complex 1 and IV, which are essential for electron transport chain and ATP synthesis essential complexes in mitochondrial function and could therefore affect sperm function.

Similarly, genes associated with inflammatory processes were also upregulated in Brn-3b KO testes. This included increased prostaglandin E synthase (*Ptges or Pges*), which is associated with production of pro-inflammatory prostanoid, prostaglandin E_2_ (Pge2)(44) and the secretory prolactin inducible glycoprotein, Pip binds to Fc fragment of immunoglobulin (IgG) and anti-sperm antibody(45).

Loss of *Brn-3b* also caused increased expression of different proteases involved in ECM remodelling, including the matrix metalloproteinase proteases, *Mmp7*, linked to high sperm DNA fragmentation and apoptosis(46). Increased expression of *Fgf10* in mutant testes suggests deregulation of signalling pathways(47). Taken together, RNA sequencing and validation studies confirmed that loss of Brn-3b affected multiple groups of target genes that are implicated in testes development, spermatogenesis and sperm function.

### Brn-3b/POU4F2 expression in human testes using HISTA single cell testis atlas:

*Brn-3b* expression has been reported in human testes(26, 29–31) but its relevance in male fertility is still not clear. Therefore, detailed analyses were next undertaken to analyse *Brn-3b* expression in human testes using the Human Infertility Single-cell Testes Atlas (HISTA) sequencing atlas containing data from 12 human donors (including testes biopsies from 6 normozoospermic adult human donors). This analysis revealed that *Brn-3b* mRNA is most highly expressed in pachytene spermatocytes (Fig. 5A). Notably, when comparing *Brn-3b* mRNA expression patterns, we observed significantly higher expression levels in testis biopsies from normozoospermic controls compared to samples from infertile men, including individuals with non-obstructive azoospermia (INF1), a case of retrograde ejaculation (INF2), and patients with Klinefelter syndrome (KS). Moreover, expression levels were also significantly higher in controls compared to testis samples from juvenile males (Fig. 5B). These expression patterns are consistent with the expected depletion or absence of meiotic and post-meiotic cells in these subjects.

Further investigation utilised the HISTA Structured Dimensionality Reduction (SDA) components to provide deeper insights into biological processes associated with *Brn-3b*. SDA is a method of soft clustering that identifies sets of co-expressed genes, which we call “components”, often corresponding to genes regulated by the same transcription factors and involved in the same biological processes(35, 48). *Brn-3b* was identified as a significant contributor to multiple SDA components, exhibiting a strong negative loading on SDAV141 (−0.404) and SDAV98 (−0.152), alongside positive loadings on SDAV21 (+ 0.191), SDAV71 (+ 0.096), SDAV74 (+ 0.094), and SDAV94 (+ 0.064). Gene Ontology (GO) enrichment analysis of these components elucidated their biological relevance in relation to *Brn-3b*. The most intriguing component involving *Brn-3b* is SDAV98 (Table 3). Strikingly, the top two negative gene loadings on this component are *Brn-3b* (*POU4F2*) and *POU5F2*, a POU family transcription factor and a paralog of *POU4F2*. Of the top 60 genes in this component, 39 are lncRNAs, and 4 are uncharacterized putative protein-coding genes (*C2orf78, C9orf57, C9orf163, C17orf96*). Most of the top 60 genes are poorly characterized, and several have been recently linked to human male infertility, including *C2orf78*, *POTEJ*, and *PROK2*.(31) GO enrichments for this component all relate to plasma membrane cell-cell adhesion (Table 3).

For SDAV141, whose negative loading correlates with *Brn-3b*/*POU4F2* expression, the enriched GO terms from its negative loadings again indicate “cell-cell adhesion”, while positive (anticorrelated) gene loadings related to ‘Regulation of mitotic cell cycle phase transition,’ and “G2/M transition of mitotic cell cycle”, perhaps suggesting that *Brn-3b* expression may be coupled to a checkpoint or cell fate decision during meiosis. More broadly, GO term enrichment across the components revealed that *Brn-3b* is co-expressed with genes involved in late spermatogenic stages, encompassing functions such as ‘calcium-dependent cell-cell adhesion via plasma membrane adhesion,’ ‘male gamete generation,’ and ‘(single) fertilization.’

Given the molecular function of *Brn-3b* as a transcription factor, it is of interest to understand what genes might be activated by this protein in male germ cells. Pseudotime trajectory analysis of germ cells demonstrated that known *Brn-3b* targets, including *SLC2A4* (encoding GLUT4) and *SHH* (Sonic Hedgehog), were expressed in development steps just downstream of *Brn-3b* expression (Fig. 5D). Like *Brn-3b*, both genes have high loading on SDAV21, identifying a possible role for *Brn-3b* in the regulation of SDAV21 genes more broadly. GO categories represented in this component include “regulation of hormone secretion” and “sperm capacitation”. These patterns are consistent *Brn-3b* playing a causal role in the upregulation of wide range of downstream effectors during spermatogenesis.

### Identification of POU4F2/Brn-3b variants associated with male infertility:

To identify potentially deleterious genetic variants in the *Brn-3b* gene that could contribute to human male infertility, we analysed whole exome sequencing (WES) data from a large international cohort sequenced as part of the ongoing GEMINI project (31, 36).

After filtering, we identified one patient affected by non-obstructive azoospermia (NOA) carrying biallelic variants in *Brn-3b*. Specifically, this patient carried two deleterious missense variants with PHRED-scaled CADD scores greater than 20 (Fig. 6). These were validated to be in trans; one of variant was located within the POU domain (Fig. 6). Additionally, among nine other patients (six with NOA and three with extreme oligozoospermia), as well as seven normozoospermic controls, we observed heterozygous, rare, and predicted to be pathogenic *Brn-3b* variants (supplementary table 1). At present, one biallelic variants seem to be a strong contributor to the infertile phenotype while further studies will be necessary to determine the relevance of other *Brn-3b* SNVs in spermatogenesis and infertility.

## DISCUSSION

The rising global burden of male infertility highlights an urgent need to elucidate the intricate molecular mechanisms governing sperm production and fertility(2, 3). Despite advances, complex aspects of spermatogenesis remain poorly understood, hindering diagnosis and treatment. Our study uses a multifaceted approach to uncover essential roles for the Brn-3b transcription factor in regulating spermatogenesis and male fertility. We demonstrate a strong link between reduced *Brn-3b* expression and infertility in both mouse models and human subjects.

*Brn-3b* mRNA and protein are predominantly expressed in mature spermatids in adult murine testes, with low levels in immature, juvenile testes(14). Key roles in late-stage spermatogenesis is further supported by loss of Brn-3b in infertile CREM mutants, characterised by arrested spermatid development(14). However, its direct role in testicular development and function has remained poorly defined. Although infertility in male homozygous *Brn-3b* KO mice was previously observed, the underlying mechanisms driving these phenotypes were unknown.

Our comprehensive analysis of the constitutive *Brn-3b* KO model reveals critical cellular and molecular alterations including pronounced structural and morphological abnormalities in developing spermatids, correlating with low sperm counts and reduced sperm motility. TEM images of *Brn-3b* KO sperm displayed ultrastructural abnormalities, affecting the sperm head (disrupted acrosomal membranes); midpiece (mitochondrial disorganisation and fragmentation) and sperm tail (structural abnormalities in the anterior portion of ODFs and fibrous sheath). These defects mirror those seen in infertile individuals and suggest underlying bioenergetic and functional deficiencies(36, 49, 50).

Transcriptomic analysis of *Brn-3b* KO mouse testes versus WT controls revealed key molecular mechanisms underlying the observed phenotypic changes. For instance, functional annotation and pathway analyses showed that loss of *Brn-3b* significantly disrupted gene ontology pathways linked to male gamete generation, spermatogenesis, mitochondrial function, and sperm motility, including microtubule motor activity. Pathways involved in sperm–egg plasma membrane fusion were also deregulated, aligning with acrosomal defects seen in TEM data. The acrosome, a cap-like organelle at the sperm head, contains hydrolytic enzymes essential for penetrating the outer layers of the egg during fertilization. Disruption or malformation of acrosomal membranes may cause premature or ineffective acrosome reactions that impairs sperm-egg binding and preventing successful fertilization(8), as seen in globozoospermia, a known cause of male infertility(50).

Spermatogenesis relies heavily on normal mitochondrial function, which supports essential processes such as sperm motility, capacitation, and the acrosome reaction. This is reflected in the ordered mitochondrial sheath of mature spermatids in fertile sperm(51) and well-documented links between mitochondrial dysfunction and impaired fertilisation(49). RNA sequencing of *Brn-3b* KO testes revealed significant disruption of mitochondrial pathways, consistent with ultrastructural abnormalities including fragmentation and disorganisation around the mid-piece and correlating with reduced motility in *Brn-3b* KO mutants.

Importantly, pathway analysis of *Brn-3b* KO mouse testes revealed significant disruption of mitochondrial pathways, particularly those linked to oxidative phosphorylation, ATP synthesis and respiratory chain activity. These changes are critical given the reliance on mitochondrial function for spermatogenesis and sperm activity. The high mitochondrial density in the sperm mid-piece underscores its central role in motility and fertilisation(9). Beyond ATP production, mitochondria also regulate steroidogenesis, cell proliferation, sperm–egg interaction via ROS signalling, and apoptosis(9, 51). Given these diverse roles, intact mitochondrial function is essential for male fertility.

qPCR validation studies showing upregulation of NDUF genes e.g. *Ndufa6* and reduction of *Cox7a1* in independent *Brn-3b* KO testes confirms disruption of key genes associated with mitochondrial complexes 1 and IV, which are key components of the electron transport chain and ATP synthesis.

His *NDUF* (NADH:ubiquinone oxidoreductase) genes encode essential components of mitochondrial Complex I, which drives ATP synthesis via proton gradient formation. Critical components of the proton translocation module within mitochondrial Complex I, (51), which is essential for generating the proton gradient that drives ATP synthesis. Overexpression of key subunits such as *Ndufa6*, while not a catalytic subunit, can disrupt the stoichiometry and functional integrity of Complex I and impair the efficiency of electron transfer and disrupt the electron transport chain. Similarly, downregulation of Cox7a1, which encodes a subunit of cytochrome c oxidase (COX; Complex IV), may compromise completion of the electron transport chain. As Complex IV functions as the terminal enzyme responsible for transferring electrons to molecular oxygen, its dysfunction can severely impair oxidative phosphorylation, leading to reduced ATP production and elevated reactive oxygen species (ROS) (52, 53).

Such disruption of key complexes within the mitochondrial electron transport chain in *Brn-3b* KO testes is therefore likely to contribute to diminished ATP synthesis, which in turn affects energy-dependent processes such as flagellar movement essential for sperm motility, as well as capacitation and the acrosome reaction which are both critical for sperm-egg fusion and successful fertilisation. These functional impairments align with the cellular pathways disrupted in *Brn-3b* KO testes.

Moreover, perturbation of the electron transport chain and increased electron leakage are strongly associated with abnormally elevated ROS production and resultant oxidative stress, commonly associated with sperm damage and male infertility(51). This is consistent with the ultrastructural abnormalities observed revealed by TEM imaging of *Brn-3b* KO sperm, including acrosomal and flagellar damage and mitochondrial fragmentation, associated with sperm damage and infertility(54) (43). While the precise mechanisms by which Brn-3b regulates mitochondrial processes in sperm remain to be fully elucidated, current findings suggest that impaired mitochondrial function in *Brn-3b* KO testes compromises the bioenergetic capacity of spermatozoa required for motility and fertilisation. Such dysfunctions likely contribute to infertility phenotypes such as asthenozoospermia (reduced sperm motility) and other forms of male infertility(49, 51).

The disruption of mitochondrial function and oxidative phosphorylation observed in male Brn-3b knockout (KO) models is particularly striking in light of the cardiovascular dysfunctions consistently reported in these mutants(16, 19). Cardiomyocytes rely heavily on fatty acid substrates to sustain contractile and bioenergetic demands, making them especially vulnerable to mitochondrial perturbations(55). The loss of Brn-3b appears to compromise mitochondrial integrity across multiple tissues, suggesting a previously unrecognized and essential role for this transcription factor in regulating bioenergetic pathways critical for cellular homeostasis. These findings not only highlight the tissue-specific importance of Brn-3b in maintaining metabolic resilience but also suggest a potential mechanistic link between male infertility and increased cardiovascular disease (CVD) risk. The convergence of mitochondrial dysfunction in reproductive and cardiac tissues may reflect a shared Brn-3b-dependent mechanism for understanding the molecular basis of these interconnected conditions in future studies to understand the molecular basis linking reproductive and cardiovascular health.

Deregulation of genes associated with structural proteins also corroborate the ultrastructural defects observed in the fibrous sheath and outer dense fibres of *Brn-3b* KO sperm flagellum. The validated down-regulation of structural genes encoding dynein heavy-chain motor proteins such as *Dnah6* in *Brn-3b* KO mutants are consistent with impaired motility observed in *Brn-3b* KO sperm. Such structural proteins are essential for cilia motility and synchronised flagellar beating necessary for progressive motility and disruption in expression or function can contribute to asthenozoospermia(38, 56, 57). Similarly, confirmed reduction of other spermatogenesis-associated genes such as *Spata13*, linked to cell migration and adhesion, may potentially affect sperm motility and fusion capability(58) while down-regulation of as *Misp3* (involved in spindle formation and cell cycle progression)(41) suggest broader disruptions in germ cell development. The combination of structural disruption in sperm tail accessory structures involved in cytoskeletal architecture and motility strongly implicates Brn-3b in preserving the structural and functional integrity of the sperm tail.

Interestingly, *Brn-3b* KO testes exhibit upregulation of inflammatory mediators including prostaglandin E synthase (*PTGES or PGES*), which catalyses the synthesis of pro- inflammatory prostanoid, PGE2(44). Additionally, elevated expression of the secretory prolactin inducible glycoprotein, *Pip*, in *Brn-3b* KO testes is noteworthy because of its ability to bind to Fc fragment of IgG and anti-sperm antibody, suggesting a potential role in immunological dysregulation(45). Given the established link between chronic inflammation and male infertility, up-regulation of such genes may represent either compensatory responses to defective spermatogenesis or contribute directly to unfavourable microenvironment for developing germ cells, thereby contributing to infertility(54, 59). Furthermore, loss of *Brn-3b* also affected expression of proteases involved in ECM remodelling, with confirmed increases in matrix metalloproteinase proteases, *Mmp7*, which is implicated in sperm DNA fragmentation and apoptosis(46) while increased expression of *Fgf10* in mutant testes suggests deregulation of signalling pathways(47). These changes are likely due to apoptosis of malformed spermatids and results in lower number of elongated spermatids as shown in Fig. 1.

Comprehensive analyses of *Brn-3b* KO testes have provided mechanistic insights linking loss of *Brn-3b* with defective spermatogenesis, characterised by abnormalities in sperm morphology, bioenergetic capacity and functional integrity. Although whole genome sequencing of testes from WT and Brn-3b KO mice must be interpreted with caution based on issue heterogeneity and potential bias in cellular composition in mutant testes, the observed molecular changes are consistent with infertility phenotype seen in male homozygous *Brn-3b* KO mutants. These findings reinforce the role of Brn-3b in maintaining reproductive function and provide further evidence of the morphological, genetic and functional disruptions that result from loss of Brn-3b.

Since Brn-3b is highly conserved between humans and mice, sharing more than 98% amino acid homology, our data from the Brn-3b KO mutant mice could have strong translational relevance for understanding the mechanisms of spermatogenesis in humans and understanding the mechanisms and basis of male infertility in the clinical setting. Multiple reports of Brn-3b/POU4F2 expression in human testis(26–29) and identification of Brn-3b/POU4F2 as a gene of interest in male fertility(29–31) is also supported our data from analysis of single-cell RNA sequencing data from the HISTA database, which show strong *POU4F2* (*Brn-3b*) expression in pachytene spermatocytes from fertile men. These findings are consistent with mouse studies that demonstrate Brn-3b protein expression in both spermatocytes and spermatids from wild-type (WT) mice and its absence in CREM knockout (KO) mutants, which lack this cell population(14).

In line with the infertility observed in *Brn-3b* knockout mice, scRNA-seq data from human testes also reveal significantly reduced populations of *POU4F2* (*Brn-3b*)-expressing cells in infertile men diagnosed with azoospermia and Klinefelter syndrome. These reduced levels are comparable to the low *POU4F2* (*Brn-3b*) expression seen in immature juvenile testes, which can be primarily attributed to the lack or substantial reduction of spermatocytes and spermatids in these conditions compared to fertile controls. Furthermore, gene ontology (GO) term enrichment analysis demonstrates that *POU4F2* (*Brn-3b*) expression significantly correlates with other genes crucial for late spermatogenic stages and fertilization. Moreover, its expression dynamics align with existing literature, as pseudotime analysis confirms that known POU4F2 targets, such as *SLC2A4* (*GLUT4*) and SHH, are expressed immediately after *POU4F2* (*Brn-3b*) expression along the spermatogenic trajectory.

Taking all this evidence together, it strongly suggests that *POU4F2* may also play a crucial role in human male infertility. To investigate this, we performed a targeted search for rare and potentially pathogenic variants in a cohort of 1,474 men with idiopathic azoospermia/ oligozoospermia and 633 normozoospermic controls, sequenced within the GEMINI cohort. This analysis revealed a potential genetic cause of human male infertility in a patient with Non-Obstructive Azoospermia, who carries two rare missense variants in *POU4F2* (*Brn-3b*). One variant was inherited from the father and the other from the mother, both exhibiting high CADD scores, indicating their likely deleteriousness. Although the functional consequences of these variants remain to be investigated, these findings, combined with data from Brn-3b knockout (KO) mutant mice, may offer important leads for understanding the genetic basis of male infertility in future studies.

In conclusion, this study provides strong evidence supporting a critical role for Brn-3b in spermatogenesis and male fertility. Compelling evidence from Brn-3b knockout (KO) mouse models has highlighted the essential roles of Brn-3b in controlling spermatogenesis, sperm development, and function. The observed infertility in Brn-3b knockout mice, coupled with single-cell RNA sequencing (scRNA-seq) data revealing significantly reduced populations of *POU4F2* (*Brn-3b*)-expressing cells in infertile men with azoospermia and Klinefelter syndrome, highlights its importance in human spermatogenesis. Furthermore, the identification of rare and potentially pathogenic *POU4F2* (*Brn-3b*) missense variants in an infertile man with Non-Obstructive Azoospermia provides compelling human genetic evidence supporting its etiologic role. These combined lines of evidence underscore the urgent need for future investigations into the functional consequences of *POU4F2* (*Brn-3b*) variants in the context of human male infertility. Ultimately, a deeper understanding of the genetic and molecular contributions of *POU4F2* (*Brn-3b*) could pave the way for novel diagnostic, prognostic, or therapeutic strategies to address male infertility and improve reproductive outcomes for affected couples.

## Supplementary Material

Supplementary Files

This is a list of supplementary files associated with this preprint. Click to download.

- SupplementaryFigure1.tif
- SupplementaryTable1ii.tif
- SupplementaryTable1i2.tif
- Table1.tif
- Table2.tif
- Table3.tif

## Data Availability

The datasets generated during and/or analysed during the current study are available in the mouse database are available from the corresponding author upon reasonable request. Datasets used for the human studies are available as follows: Human Infertility Single-cell Testis Atlas (HISTA) repository: https://conradlab.shinyapps.io/HISTA/ Whole exome sequencing (WES) data from GEnetics of Male INfertility Initiative (GEMINI): https://gnomad.broadinstitute.org/
